# The construction of a testis transcriptional cell atlas from embryo to adult reveals various somatic cells and their molecular roles

**DOI:** 10.1186/s12967-023-04722-2

**Published:** 2023-11-27

**Authors:** Najmeh Salehi, Mehdi Totonchi

**Affiliations:** 1 School of Biological Science, Institute for Research in Fundamental Sciences (IPM), Tehran, Iran; 2 Department of Genetics, Reproductive Biomedicine Research Center, Royan Institute for Reproductive Biomedicine, ACECR, Tehran, Iran

**Keywords:** Single-cell RNA sequencing, Male infertility, Somatic cells, Weighted gene co-expression network analysis, Heterogeneity, Paracrine cell–cell communication

## Abstract

**Background:**

The testis is a complex organ that undergoes extensive developmental changes from the embryonic stage to adulthood. The development of germ cells, which give rise to spermatozoa, is tightly regulated by the surrounding somatic cells.

**Methods:**

To better understand the dynamics of these changes, we constructed a transcriptional cell atlas of the testis, integrating single-cell RNA sequencing data from over 26,000 cells across five developmental stages: fetal germ cells, infants, childhood, peri-puberty, and adults. We employed various analytical techniques, including clustering, cell type assignments, identification of differentially expressed genes, pseudotime analysis, weighted gene co-expression network analysis, and evaluation of paracrine cell–cell communication, to comprehensively analyze this transcriptional cell atlas of the testis.

**Results:**

Our analysis revealed remarkable heterogeneity in both somatic and germ cell populations, with the highest diversity observed in Sertoli and Myoid somatic cells, as well as in spermatogonia, spermatocyte, and spermatid germ cells. We also identified key somatic cell genes, including RPL39, RPL10, RPL13A, FTH1, RPS2, and RPL18A, which were highly influential in the weighted gene co-expression network of the testis transcriptional cell atlas and have been previously implicated in male infertility. Additionally, our analysis of paracrine cell–cell communication supported specific ligand-receptor interactions involved in neuroactive, cAMP, and estrogen signaling pathways, which support the crucial role of somatic cells in regulating germ cell development.

**Conclusions:**

Overall, our transcriptional atlas provides a comprehensive view of the cell-to-cell heterogeneity in the testis and identifies key somatic cell genes and pathways that play a central role in male fertility across developmental stages.

**Supplementary Information:**

The online version contains supplementary material available at 10.1186/s12967-023-04722-2.

## Background

Testis undergoes significant changes in both physiology and morphology from fetal life to adulthood, driven by complex hormonal and molecular adjustments that promote the maturation of somatic cells and the initiation of spermatogenesis [1, 2]. The primordial germ cells, which are the primary undifferentiated stem cells, migrate to the gonadal ridge during embryonic development and differentiate into gonocytes in male embryos [3]. These gonocytes, along with other embryonic precursors of sperms called fetal germ cells (FGCs) [4], eventually give rise to spermatogonial stem cells (SSCs) after birth [5]. SSCs have the unique ability to self-renew, maintaining the stem cell pool throughout life while also differentiating into spermatocytes (SPCs) through active mitosis. SPCs undergo two meiotic cell divisions, giving rise to haploid cells called spermatids (SPTs), which differentiate into mature sperm through the complex process of spermatogenesis [6]. Somatic cells such as Sertoli cells, peritubular myoid cells, and Leydig cells play an essential role in testis formation and spermatogenesis [7, 8]. However, our understanding of somatic cell heterogeneity and their precise roles in spermatogenesis remains limited.

Male fertility and puberty rely on spermatogenesis within the seminiferous epithelium of the testis to facilitate the production of millions of sperm in normal men [9, 10]. Regrettably, 17.5% of couples worldwide face infertility, and half of these cases are related to male factors [11]. Male infertility is typically caused by spermatogenesis abnormalities with unknown causes, making it difficult to treat [12, 13]. Meanwhile, between 1500 to 2000 genes play a role in spermatogenesis, and any changes in these genes could disturb male fertility [10, 14]. Genetic diagnosis of male infertility involves screening a small number of candidate genes, mainly based on case reports [13, 15]. High-resolution profiles of gene expression in spermatogenesis can disclose new insights and identify many key genes involved in this process [16].

Single-cell RNA sequencing (scRNA-seq) approaches can provide high-resolution profiles to explore the difference within and between cell types, find rare cell types, track the trajectories of cells in development, uncover the function of each cell type, and show regulatory relationships between genes [17]. In this regard, scRNA-seq analysis of FGCs and their gonadal niche cells in male and female human embryos was performed, revealing multiple developmental stages [4]. In some studies, alterations in spermatogenic cell types were evaluated during age changes [2, 18–20]. Most of the scRNA-seq studies investigated the process of spermatogenesis in fertile adult men [2, 18–23]. However, few studies have explored scRNA-seq in men with infertility disorders [24–26]. All of these studies have attempted to find heterogeneity between and within spermatogenic cell types by clustering, identifying differentially expressed genes (DEGs), and performing enrichment analysis.

Studies have demonstrated the essential role that somatic cells play in promoting spermatogenesis. These cells facilitate complex cell-to-cell interactions, transport proteins, express crucial genes in the spermatogenesis process, and produce enzymes and regulatory factors [27–31]. However, most studies have focused on the various types of Sertoli cells and their interactions with germ cells [2, 26, 32–35] with less attention given to other somatic cells. Therefore, a thorough investigation of somatic cells is necessary to fully comprehend the effects and contributions of these cells on spermatogenesis. So, in this study, we constructed a transcriptional cell atlas of the testis from embryo to adult to gain a more comprehensive understanding of somatic cell diversity and their contributions to male fertility. To characterize cell heterogeneity in this atlas, various analyses including clustering, cell type assignments, identification of DEGs, enrichment analysis, and pseudotime trajectory analysis were conducted. In addition, a gene co-expression network and cell–cell communication network were generated, and their topological analysis identified important genes in somatic cells associated with male infertility in this fertile data.

## Methods

### Data retrieval and processing

Raw scRNA-seq data for male fetal gonads (1187 cells) were retrieved from the Gene Expression Omnibus (GEO) [36] repository through accession number GSE86146 [4]. This data was collected from twelve male embryos of 4 to 25 weeks old (W_e_) which were classified as “Fetal” data in this study. The scRNA-seq data for 2 and 7 days old, “Infancy” data, was selected from GSE124263 [19] which contains 8789 unfractionated cells from the testes of two neonatal. For the “Childhood” data collection, 1341, 1968, and 4176 cells from about 1 year old (GSE120506) [18], 7, and 11 years old (GSE134144) [2] donors were downloaded from GEO. Also, the scRNA-seq data of 13- and 14-years old donors in the GSE134144 data series containing 4051 and 2722 cells, respectively, were used for “Peri-puberty”. To have a comprehensive comparison, the steady state spermatogenic cells of GSE109037 and GSE106487 data series were gathered in this study with 7134 and 3046 cells, respectively [21, 22]. The dataset names, age of donors, scRNA-seq methods, GEO ID, and the initial number of cells in each dataset were summarized in Additional file 1: Table S1. The Seurat4.0.0 R package [37] was used for scRNA-seq data analysis. In each data, cells with less than 500 expressed genes and genes with less than 3 cells expression were removed. Also, cells with a high or low number of genes, and cells with more than 25% of mitochondrial genes were filtered. For each data, the “LogNormalize” method was used for normalization and highly variable features were identified. Finally, 26,642 cells were gathered for integration.

### Data integration and analysis

In order to relate different data from different groups of donors and across different scRNA-seq technologies, the anchor strategy was used. In this strategy, an unsupervised method is implemented to find an anchor set with a common biological state for data integration. 35 dimensions were used in the anchor weighting procedure. Then a linear transformation (scaling), and dimensional reduction (principal component analysis (PCA)) were performed on the new integrated dataset. The Uniform Manifold Approximation and Projection (UMAP) [38], as a non-linear dimensional reduction technique, was used to visualize and explore this integrated dataset. The number of dimensions was set to 35 according to the ranking of principal components based on the percentage of variance, and other parameters of UMAP implementation were set by default. To cluster the cells, the graph-based clustering approach implemented in the Seurat R package was used. The dimensionality and the resolution parameters to construct a shared nearest neighbor graph and clustering were set to 35 and 0.3, respectively. The specific markers of testicular germ and somatic cells were collected from the literature and evaluated to assign the cell type of clusters.

### Differentially expressed genes and enrichment analysis

The integrated dataset was used to find DEGs between different clusters. The Seurat R package uses the non-parametric Wilcoxon rank sum test [39] to identify the positive and negative markers of each cluster compared to all other clusters. The minimum percentage (min.pct) and the log fold-change of the average expression (logfc.threshold) were set to 0.25 and 0.5, respectively. The Database for Annotation, Visualization, and Integrated Discovery (DAVID) v6.8 [40] was used for gene enrichment analysis. The up-regulated genes of somatic cells with an averaged log fold-change > 0.7 and adjusted p-value (based on Bonferroni correction) < 0.05 were considered for enrichment analysis. The biological processes (BPs) terms were filtered based on the Benjamini correction score (adjusted p-value).

### Pseudotime analysis

Monocle3 R package was used for pseudotime analysis [41]. In this regard, the integrated data, dimension reduction, and clustering information were imported from Seurat to the Monocle3 package. Monocle learns the sequence of gene expression changes of each cell as part of a dynamic biological process and places each cell at its proper position in the trajectory.

### Co-expression network construction and analysis

To identify groups of genes that tend to be expressed together in a coordinated manner across various cells and developmental stages, a weighted gene co-expression network (WGCN) was created by the WGCNA R package [42]. Various values of soft thresholding power β were evaluated to create the WGCN with a scale-free topology. The value of 5 was ultimately chosen. The Pearson correlation coefficient was used to measure the correlation between the expression of each pair of genes and the signed network option was used to maintain only positive correlations. The topological overlap measure (TOM) was used to uncover modules in the WGCN. This measure assesses the similarities between gene pairs by counting the number of shared neighbors in the network. The modules were visualized in the network by assigning different colors to each group of co-expressed genes. Any genes that lack significant co-expression with other genes were placed in a gray module and were removed from further analysis. The relationships between the different modules were illustrated by utilizing module eigengenes, which represent the first principal component of gene expression within each module. Constructed WGCN was exported to Cytoscape [43] and CytoNCA [44] was used for centralities measurements. To measure the p-value for each gene, the random gene label permuting was used for 100,000 steps.

### Cell communication inference

The SingleCellSignalR R package [45] is utilized to infer intercellular signaling communications. This approach profiles the ligand-receptor (L-R) interaction from scRNA-seq data based on a comprehensive database of known L-R interactions using a new regularized product score. The paracrine interactions between cell clusters were computed, and the s.score (LRscore) and logFC were set to 0.7 and 1.5, respectively. The cell–cell communication network was exported to Cytoscape [43] and CytoNCA[44] was used for centralities measurements.

## Results

### Testis transcriptional cell atlas from embryo to adult.

**Fig. 1:**
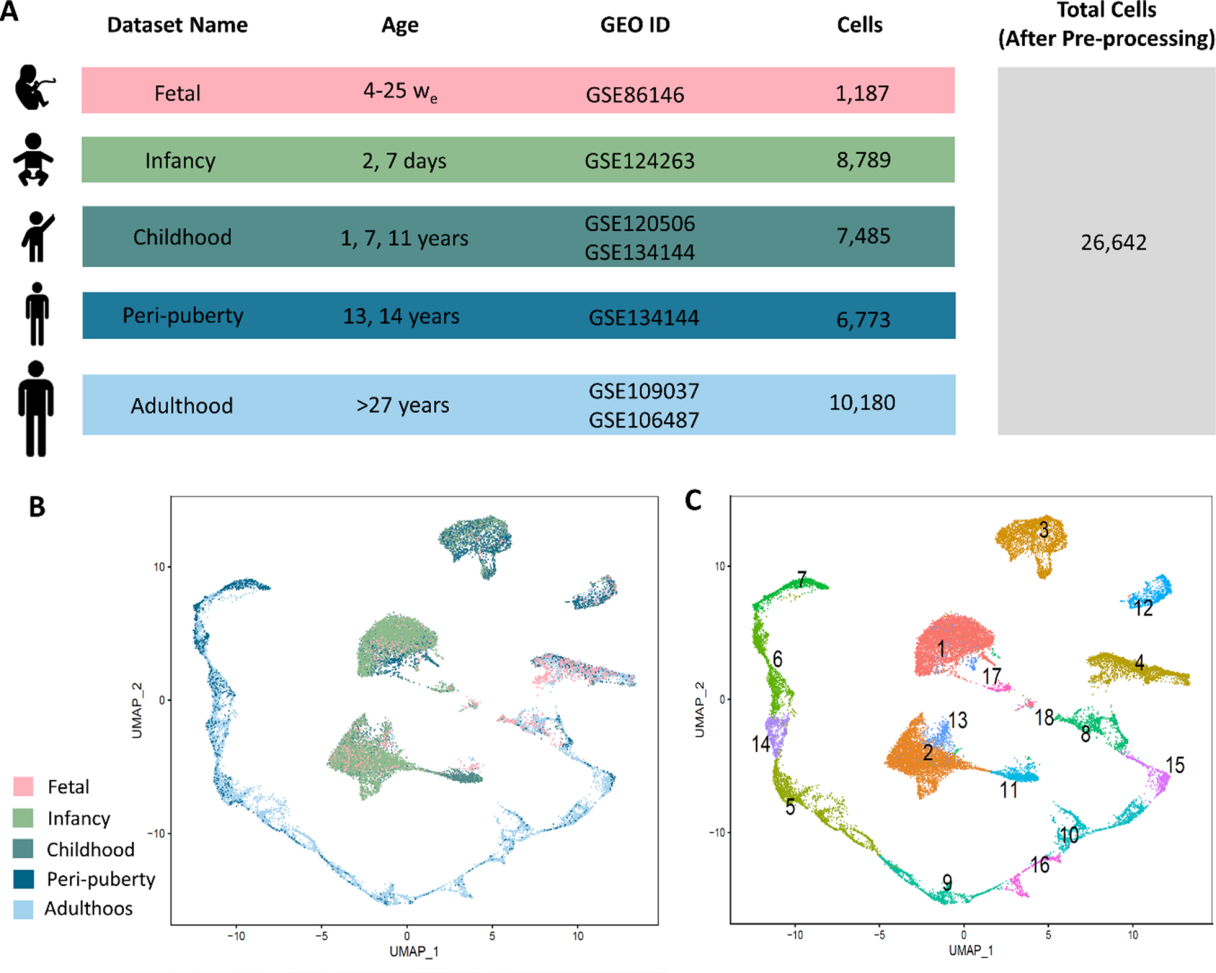
Profiling and integrating scRNA-seq data of male fetal gonad cells to adult testis cells. **A** Datasets information such as name, age, GEO ID, the number of cells for each data set, and total cells were listed. **B**, **C** UMAP plot of integrated testis transcriptional cell atlas from male fetal gonad cells to adult testis cells. Cells are colored based on **B** the classified datasets, **C** clustering results

Raw scRNA-seq data of fetal gonad cells and testis cells from individuals aged from two days to adulthood, a total of 34,414 cells, were collected from the GEO database (Fig. 1A, Additional file 1: Table S1). Each dataset was independently filtered and normalized as mentioned in the method section. These scRNA-seq data were classified into five datasets: fetal (4–25 w_e_), infancy (2, 7 days), childhood (1, 7, 11 years), peri-puberty (13, 14 years), and adulthood (> 27 years). After pre-processing, 26,642 cells were integrated to construct a testis transcriptional cell atlas from embryo to adult. Representation of the integrated datasets in the low-dimensional space of UMAP (Fig. 1B) showed that similar cells in different datasets were well-aligned, while most adult cells appeared separately and did not align with cells in other datasets, consistent with testis development. The representation of each dataset’s cells in the integrated low-dimensional space of UMAP is presented in Additional file 1: Fig. S1. Unsupervised graph-based clustering revealed 18 clusters in this integrated data, some of which are continuous and others in isolated clusters in the UMAP graph plot (Fig. 1C).

### Cell type assignment shows heterogeneity among testicular cells.

**Fig. 2:**
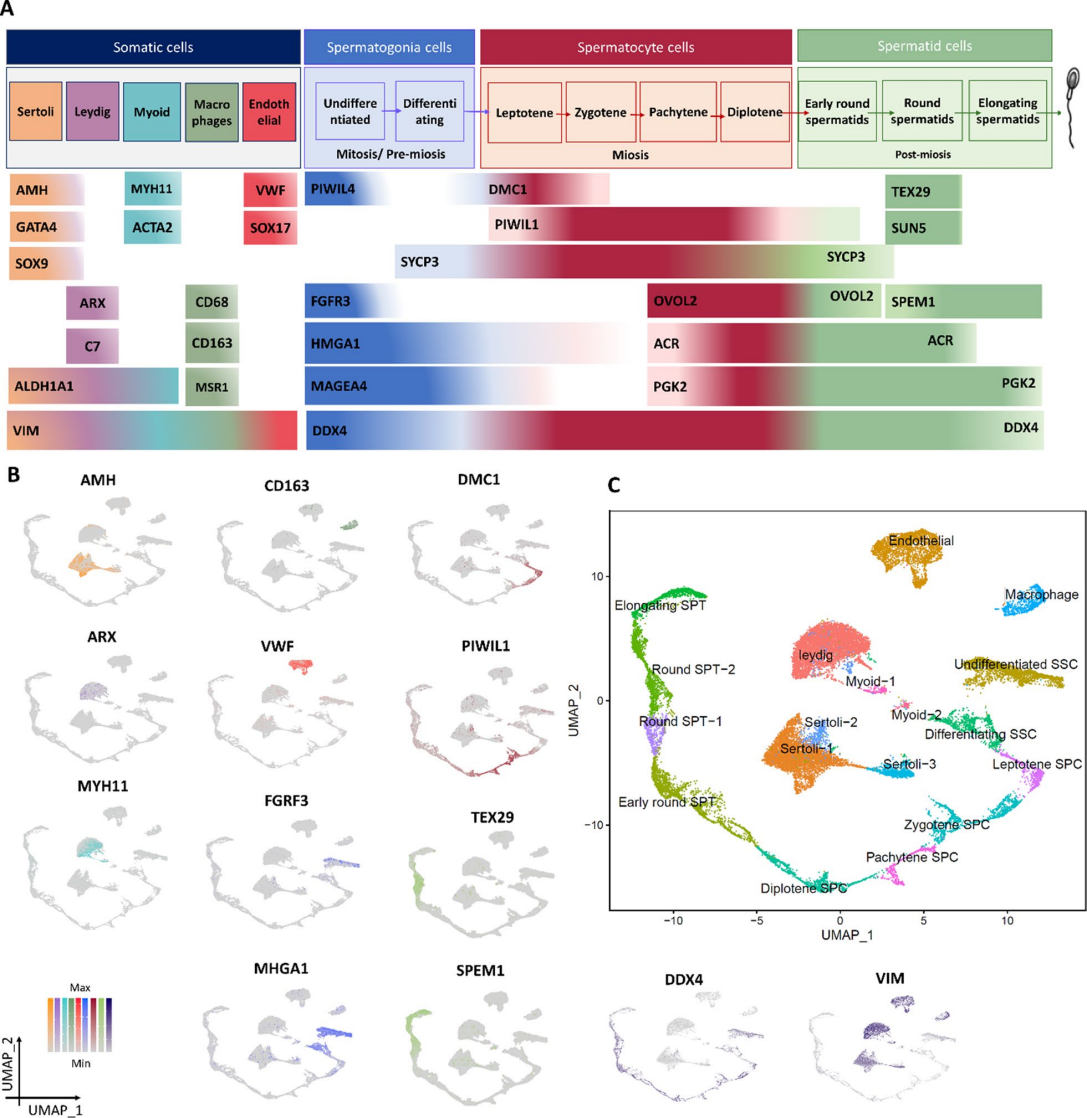
Cell type assignment of clusters. **A** Gene markers of male testis were categorized based on different somatic, spermatogonia, spermatocyte, and spermatid cells, **B** gene expression patterns of these markers were shown on the UMAP space and colored based on the A part categorization, **C** cell type assignment of clusters based on gene markers expression patterns

Known markers of somatic and germ cells were selected, and their expressions were evaluated to determine the cell type of each cluster. According to the expression pattern of *AMH*, *GATA4*, and *SOX9* [46], clusters 2, 11, and 13 correspond to the Sertoli cells (Fig. 2A, B, Additional file 1: Fig. S2). *ARX* and *C7* [4, 47] as Leydig markers were expressed in cluster 1 (Fig. 2A, B, Additional file 1: Fig. S3). Clusters 17 and 18 with high expression of *MYH11* and *ACTA2* represent myoid cells [22, 48] (Fig. 2A, B, Additional file 1: Fig. S4). *ALDH1A1* was expressed as a common marker between Sertoli, Leydig, and Myoid cells in all these clusters (1, 2, 11, 13, 17, and 18) [49] (Additional file 1: Fig. S3). Macrophage (*CD68*, *CD163*, and *MSRC1*) [50, 51] and Endothelial (*VWF* and *SOX17*) [18, 52] markers showed high expression in Cluster 12 and 3, respectively (Fig. 2A, B, Additional file 1: Figs. S5, S6). *PIWIL4* is one of the key genes for SSC maintenance expressed in cluster 4 [18]. *MAGEA4* and *HMGA1* are markers for undifferentiated and differentiating SSCs expressed in both clusters 4 and 8, indicating cluster 8 as differentiating SSCs [22, 53] (Fig. 2A, B, Additional file 1: Fig. S7). It is worth noting that FGCs and SSCs exhibit a high degree of similarity, particularly in their gene expression patterns [54], which has led them to be grouped together in the same cluster. *DMC1* is a mitotic gene and Leptotene SPC marker [22] is expressed in cluster 15. *PIWIL1* shows the highest expression level in zygotene and pachytene SPC cells while it is expressed from spermatocyte to spermatid cells [55]. *SYCP3* expression starts from differentiating SSCs and continues to early round SPT cells [56]. *OVOL2* is a common marker between zygotene, pachytene, and diplotene SPC cells correlated to the presence of the sex body during male meiosis in mammals [57]. Thus, Clusters 10, 16, and 9 correspond to zygotene, pachytene, and diplotene SPC cells, respectively (Fig. 2A, B, Additional file 1: Fig. S8). Clusters 6 and 14 with high expression of *TEX29* and *SUN5* markers were identified as round SPT cells [22]. *ACR* and *PGK2* are markers for zygotene to round spermatids and elongating spermatids, respectively [58, 59]. *SPEM1* is expressed in both round and late SPT [60], suggesting that cluster 7 belongs to elongating SPT cells (Fig. 2A, B, Additional file 1: Fig. S9). Finally, the expression pattern of *VIM* as a somatic and *DDX4* as a germ cell marker [61, 62] confirm all these cell type assignments. These cluster assignments were depicted in the UMAP space in Fig. 2C. The number of cells from each dataset in each cluster and related cell type assignments is summarized in Additional file 1: Table S2.

**Fig. 3:**
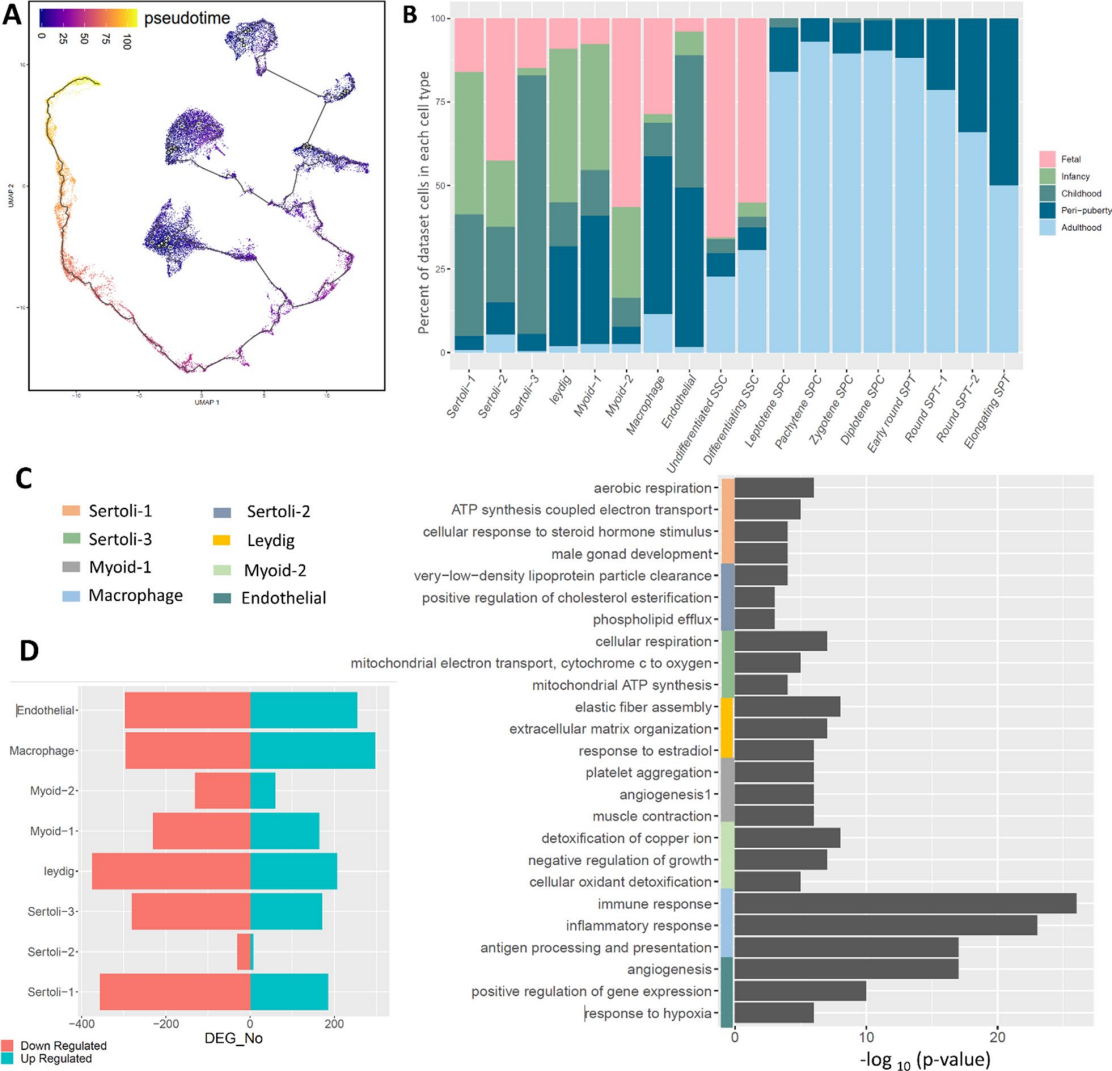
Developmental ordering of testicular cells and somatic cells enrichment. **A** The pseudotime analysis of testicular cells on the UMAP space, **B** the percent of datasets cells in each cell type, **C** the biological processes enrichment for up-regulated genes of somatic cells, **D** the number of up- and down-regulated genes in somatic cell types

The developmental timeline of all cells in the 2D UMAP space shows that the germ cell formed in an unbroken, continuous way which is consistent with the developmental order of spermatogenesis, but somatic cells were represented as independent islands (Fig. 3A). The percentage of dataset cells in each cell type showed the presence of undifferentiated and differentiating SSCs in all datasets from fetal to adulthood. However, the later developmental germ cells, SPCs, and SPTs are mainly present in the peri-puberty and adulthood datasets (Fig. 3B). Different somatic cell types were present from fetal to adulthood in various percentages. Sertoli-2, -1, and -3 are mostly present in Fetal-, Infancy-, and Childhood-related datasets, respectively. A relatively high proportion of Moyid-2 cells belong to the Fetal dataset, while this proportion is reduced for Myoid-1. These results led us to focus on somatic cells. The DEGs in all clusters were presented in Additional file 2. The top BPs of up-regulated genes in Sertoli-1 are “aerobic respiration”, “ATP synthesis coupled electron transport”, “cellular response to steroid hormone stimulus”, and “male gonad development”, while Sertoli-2 was enriched with “very-low-density lipoprotein particle clearance”, “positive regulation of cholesterol esterification”, “phospholipid efflux” and Sertoli-3 BPs were “cellular respiration”, “mitochondrial electron transport, cytochrome c to oxygen”, “mitochondrial ATP synthesis” (Fig. 3C). According to these results, the top BPs of up-regulated genes in Sertoli-1 are related to energy metabolism and male gonad development, while Sertoli-2 is enriched with BPs related to cholesterol metabolism, and Sertoli-3 is associated with BPs related to cellular respiration and mitochondrial function. Myoid-1 and -2 cells are involved in different BPs. Myoid-1 cells are involved in “platelet aggregation”, “angiogenesis”, and “muscle contraction”, while Myoid-2 cells are involved in “detoxification of copper ion”, “negative regulation of growth”, and “cellular oxidant detoxification” (Fig. 3C). The number of up- and down-regulated genes in somatic cell types indicated the most changes in Endothelial, Macrophage, Leydig, and Sertoli-1 (Fig. 3D).

### Gene co-expression network analysis uncovers cell-specific modules in spermatogenesis.

**Fig. 4:**
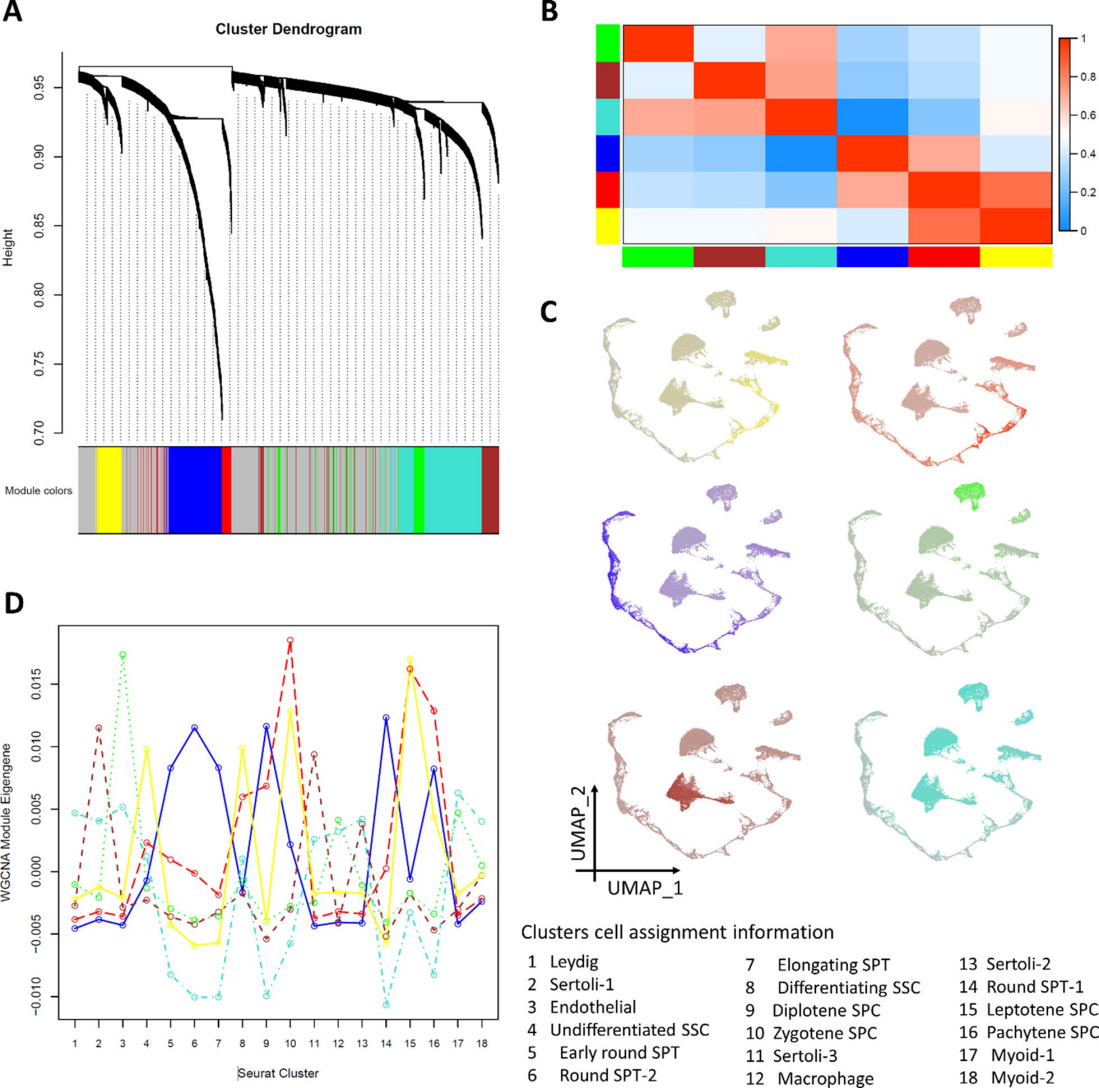
Weighted gene co-expression network analysis. **A** The clustering dendrogram of the weighted gene co-expression network (The resulting modules are depicted in different colors), **B** the eigengene adjacency heatmap displays the relationship between these modules, **C** the gene expression patterns for each module are shown on the UMAP space with their corresponding colors, **D** the eigengene of each module in each cluster

**Fig. 5:**
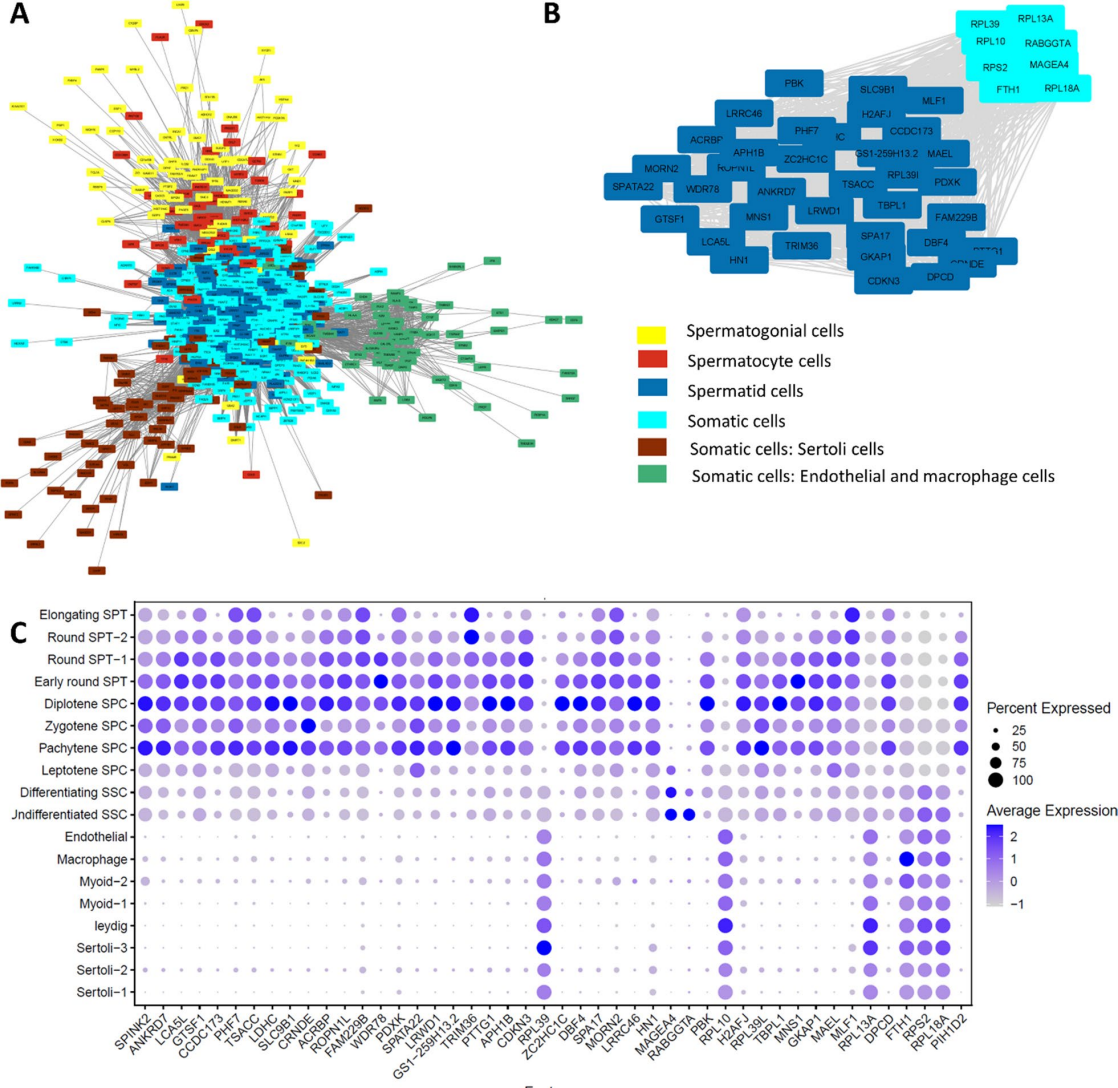
The topological analysis of the weighted gene co-expression network. **A** The presentation of the weighted gene co-expression network, **B** The subgraph of genes with the highest degree centralities. **C** The expressions of these top-degree centrality genes along different cell types

The clustering dendrogram analysis of the WGCN uncovered the presence of 6 distinct modules (Fig. 4A). The eigengene adjacency heatmap presented a strong correlation between the red-yellow, red-blue, turquoise-brown, and turquoise-green modules (Fig. 4B). The concentration of gene expression in the yellow module on the UMAP space was found to be higher in clusters 4 and 8, indicating that this module is associated with co-expressed genes in spermatogonia cells (Fig. 4C, D). The location of the red module eigengenes on the UMAP space and the presence of higher values in clusters 15, 16, 10, and 9 suggest that this module is connected to the spermatocyte cells, as shown in Fig. 4C, D. The analysis of the blue module showed a correlation between it and spermatid cells, with higher expression levels found in clusters 5, 14, 6, and 7. The green module was found to be related to the expression of genes in both endothelial and macrophage cells, while the brown module was related to the expression of genes in Sertoli cells. Meanwhile, the turquoise module comprised gene expression from all somatic cells (Fig. 4C, D). Figure 5A displays the WGCN of the integrated data using Cytoscape and is color-coded according to their related modules. The nodes that have high degree values and low p-value are identified in Additional file 3 and their expressions are shown in Fig. 5B, which belong to the blue and turquoise modules. Furthermore, the expression of these genes was assessed in various cell types, revealing that the majority of these genes were expressed in pachytene spermatocytes to elongating spermatids, while some of them were expressed in somatic cells (Fig. 5C). The top-degree genes with high expression in somatic cells were *RPL39*, *RPL10*, *RPL13A*, *FTH1*, *RPS2*, and *RPL18A*.

### Assessment of paracrine cell–cell communication exposed top ligand-receptor (L-R) interactions between somatic and germ cells.

**Fig. 6:**
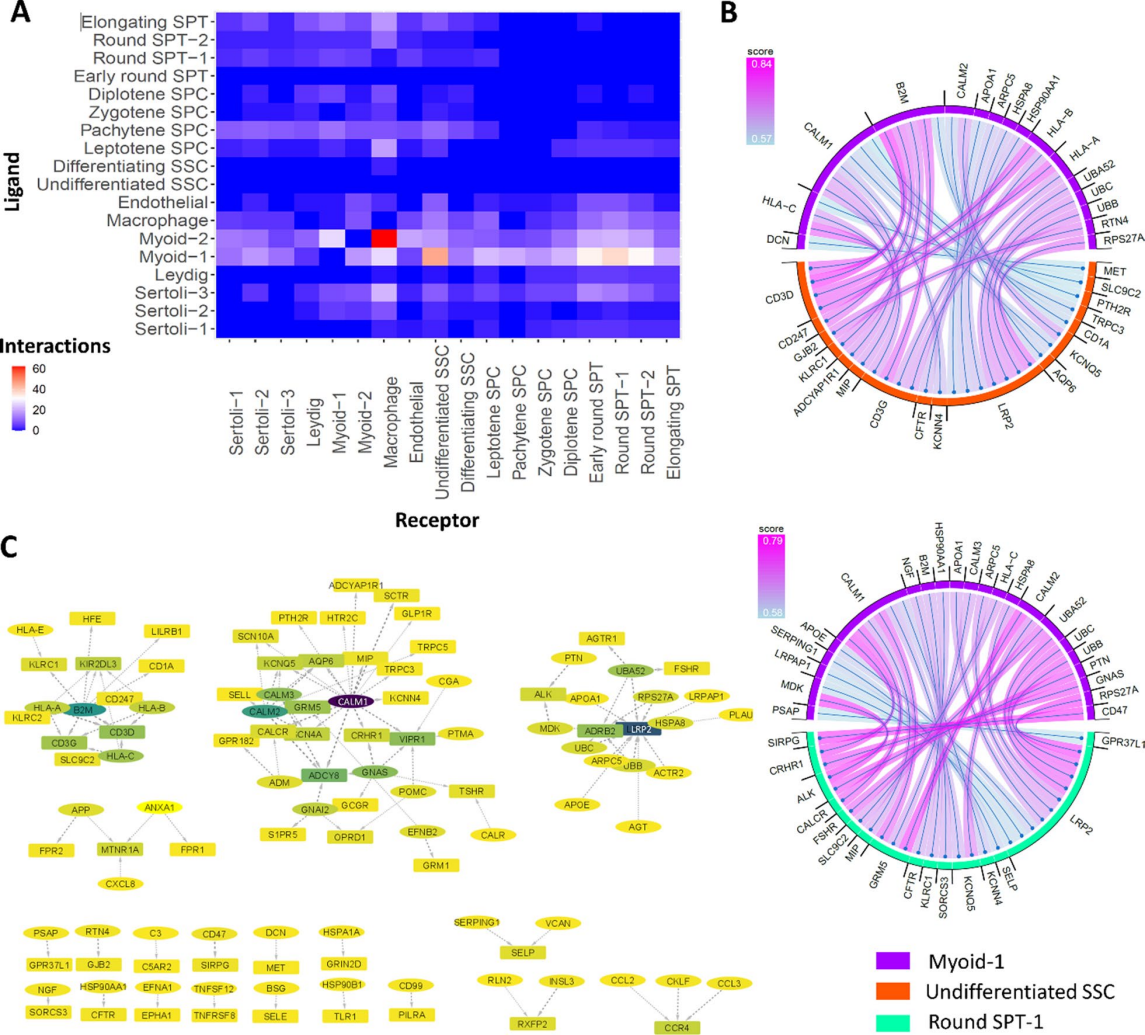
Paracrine cell–cell communication network. **A** Heatmap showing the number of ligand-receptor (L-R) interactions between different cell types. **B** L-R interactions between Myoid-1 and undifferentiated SSC, and Myoid-1 and Round SPT-1. **C** Paracrine cell–cell communication network between somatic and germ cells. Ligands and receptors are shown with oval and rectangle nodes, and they colored based on the degree from yellow to purple

Paracrine cell–cell communication was assessed among all cell types in scRNA-seq profiles (Additional file 4). The number of interactions between each pair of cells as ligand and receptor was depicted in Fig. 6A, which shows that somatic cells such as Myoid-1 and -2, Macrophage, and Sertoli-3 cells have the most ligands interacting with germ cells as the receptors. The most frequent L-R interactions were between the Myoid-1 and undifferentiated SSC and round SPT-1 (Fig. 6B). Additional file 1: Fig. S10 displays the details of other common paracrine somatic-germ cell communications, in addition to the ones mentioned in Fig. 6B. The details of all somatic and germ cells L-R interactions were reported in Additional file 5, sorted based on the LRscore. The top ten L-R pairs involving somatic-to-germ cell paracrine communication, ranked by their scores, were HLA-C-SLC9C2, CALM1-MIP, CALM2-ADCY8, CALM2-GRM5, B2M-CD3D, CALM1-ADCY8, CALM1-CRHR1, CALM1-GRM5, CALM1-SCTR, and PTMA-VIPR1. The weighted L-R interaction network between somatic and germ cells uncovered that the largest module (Fig. 6C) was enriched with the “Neuroactive ligand-receptor interaction” “cAMP signaling pathway,” and “Estrogen signaling pathway”. Additionally, the most influential nodes (hub nodes) in this weighted L-R interaction network were CALM1, B2M, CALM2, GNAS, and CALM3 as the ligand, and LRP2, ADCY8, VIPR1, ADRB2, and CD3D as the receptor (Fig. 6C, Additional file 6).

## Discussion

Male germ cell development is a complex process, encompassing various stages from FGCs to SSCs and culminating in spermatogenesis, which produces mature sperm cells. To gain a more comprehensive understanding of this process and create a transcriptional cell atlas of the testis, scRNA-seq datasets from male fetal gonad cells to adult testis cells were integrated [2, 4, 16, 18, 19, 21, 22]. These datasets were grouped into five categories based on developmental stage: fetal (4–25 weeks), infancy (2–7 days), childhood (1, 7, 11 years), peri-puberty (13, 14 years), and adulthood (> 27 years). Integrating diverse datasets from various developmental stages, even with limited sample sizes, has the potential to provide valuable insights by allowing information from one experiment to inform the interpretation of another [63]. By aligning samples across different studies using common highly variable genes, we were able to identify shared subpopulations across studies, resulting in the inclusion of similar cell types in different datasets and providing a comprehensive overview of the integrated data.

Through clustering, marker expression, and cell type assignment of integrated data, various somatic and germ cell types in the testis can be identified which are not present in individual datasets. While somatic cells provide essential support and protection to developing germ cells, our understanding of the somatic microenvironment is limited [26, 32]. Sertoli cells are the most abundant somatic cell type in the testis that play a crucial role in orchestrating spermatogenesis and supporting germ cell development, as well as regulating other somatic cells [33]. Previous studies have proposed that Sertoli cells undergo two developmental stages, pre- and post-puberty [31, 34]. However, a scRNA-seq study has reported that there are two immature Sertoli cell states that converge into a single mature state [2]. Another scRNA-seq study has also introduced three stages of Sertoli cell development, with two present during infancy and puberty and all three present in adulthood [26]. In our study, also three different Sertoli cell types were identified that were present in varying proportions across five different studied developmental stages. These variations in Sertoli cell types in different studies may be due to inadequate sampling. Interestingly, the up-regulated genes in each of the three Sertoli cell types were associated with distinct biological process sets. For example, the up-regulated genes in Sertoli-1 were primarily associated with energy metabolism, male gonad development, and steroid hormone stimuli, which is consistent with the critical role of Sertoli cells in the development and function of the testes, maintenance of male reproductive physiology, and their high metabolic activity and energy demands [35, 64, 65]. Sertoli-2 showed enrichment in biological processes related to lipid and cholesterol metabolism, which is known to be a crucial function of Sertoli cells for spermatogenesis and male fertility [66–68]. The enrichment of biological processes related to Sertoli-3 suggests that these cells may have high mitochondrial activity and energy metabolism, which is also essential for the high metabolic activity of Sertoli cells [69, 70]. Conversely, the relative abundance of Sertoli-2 cells during the Fetal development stage is closely linked to their active role in lipid and cholesterol metabolism. This suggests that these cells are actively involved in laying the foundation for future sperm production and the promotion of male fertility [71]. The enrichment of Sertoli-1 cells in biological processes, linked to their active role in energy metabolism, and their high prevalence during the Infancy development stage supports spermatogenesis and the development of the male reproductive system [72]. Additionally, Sertoli-1 cells’ role in male gonad development and responsiveness to steroid hormones emphasize their critical role in establishing and maintaining male reproductive function as the individual progresses from infancy into subsequent developmental stages [35, 73]. The present of Sertoli-3 cells during the Childhood stage, particularly in processes related to mitochondrial activity and energy metabolism, signifies their role in providing the energy necessary for growth, development, and the ongoing maturation of the male reproductive system [74]. Heterogeneity was also found among Myoid cells. Enrichment analysis indicated Myoid-1 cells play a pivotal role in muscle function and vascularization, providing structural and functional support that is essential for testis and spermatogenesis [75, 76]. On the other hand, enrichment analysis suggested Myoid-2 cells are involved in cellular detoxification and growth regulation, crucial processes that maintain cellular homeostasis and function in the testicular cells [77, 78]. These cells which are highly present during Fetal stage, are likely involved in detoxification and growth regulation, contributing to the maintenance of cellular homeostasis and the establishment of the proper cellular environment necessary for the development and function of the male reproductive system as the fetus progresses towards later stages of development [78].

A comprehensive collection of co-expression linkages among genes, which can be inferred from scRNA-seq data, is known as a gene co-expression network. It is a powerful tool to comprehend the intricate interplay between genes in biological systems [79]. By performing WGCN analysis, six modules of co-regulated genes were identified, which, when adapted to cell clusters, were attributed to the main stages of testicular cells, including all somatic, Endothelial, Sertoli, SSC, SPC, and SPT cells. This approach, known as “guilt by association,” was in agreement with the identified co-expressed gene modules [79, 80]. Identifying highly connected genes in a WGCN, also known as hub genes, can help identify essential genes within each module that are more relevant to the network's functionality than other nodes [80]. In this study, most hub genes of the WGCN were expressed in pachytene spermatocytes to elongating spermatids, with some of them also being expressed in somatic cells. These somatic hub genes included *RPL39*, *RPL10*, *RPL13A*, *FTH1*, *RPS2*, and *RPL18A*. *RPL39* is a gene that is expressed at very high levels in the testis and embryonic stem cells. It is also up-regulated in many cancer cell lines, particularly in hepatocellular carcinoma tumors [81]. Recent studies have suggested that RPL39 and its associated proteins that participate in ribosome biogenesis and protein synthesis may be correlated with human infertility [82]. Furthermore, the deficiency of *Rpl10l*, a gene with testis-specific expression, can disturb ribosome biogenesis in late-prophase spermatocytes and prohibit the transition from prophase into metaphase of the first meiotic division, resulting in male infertility [83]. Moreover, RPL13 is a potential dominant effector that can decrease sperm storage efficiency [84]. A study found significant correlations between the percentage of post-thaw motile sperm and RPL13 [85]. Additionally, *RPL18A* is identified as a differentially expressed gene in infertile endometriosis [86]. These findings suggest that protein synthesis is a critical process in these somatic cells and dysregulation of this process could lead to testicular disorders. *FTH1*, a gene encoding ferritin heavy chain protein, has been found to be down-regulated in granulosa and cervical cells of infertile women, suggesting its potential role in female infertility [87]. In contrast, *FTH1* has been shown to be overexpressed in the unilateral varicocele group compared to the fertile group, indicating its association with male infertility [88]. These findings suggest that FTH1 may play a crucial role in the regulation of reproductive processes.

By secreting signaling molecules, paracrine signaling can affect nearby cells [89]. Analyzing scRNA-seq data for the expression of ligands and receptors involved in paracrine signaling enables the identification of cell–cell interactions involved in specific biological processes [45]. Paracrine signaling is used in various cellular processes and can be dysregulated in diseases. Identifying dysregulated paracrine signaling molecules in a disease state can help identify potential therapeutic targets for drug development [90–93]. Paracrine signaling can influence cell behavior, including differentiation or migration [89, 94]. Moreover, cell-to-cell communications between somatic and germ cells are fundamental requirements of normal spermatogenesis [29]. Assessment of paracrine cell–cell communication on this scRNA-seq integrated revealed the effect of somatic cells as ligands on the germ cells as receptors. Most interactions were detected between Myoid, Macrophage, Sertoli cells with SSCs cells as the ligands and receptors, respectively. Myoid and Sertoli cells play a signaling role in the regulation of undifferentiated SSCs, thus contributing to their maintenance in male mice [76, 95]. Decoding the spermatogonial stem cell niche under physiological and recovery conditions in adult mice and humans showed niche is composed of multiple cell types, including Sertoli and Myoid cells. The interaction between SSCs and the niche is essential for maintaining SSC homeostasis [96]. An unexpected role for macrophages within the spermatogonial niche in the testis has emerged, as they express actors like CSF1 and enzymes involved in retinoic acid (RA) biosynthesis, known to influence spermatogonial proliferation and differentiation [97]. Our results showed the largest module in the L-R interaction network enriched the Neuroactive ligand-receptor interaction which plays an important role in both the nervous system and reproduction system to regulate testicular function [98, 99]. The cAMP signaling pathway is another enriched pathway that plays a crucial role in male fertility by regulating spermatogenesis, sperm motility, and capacitation [100, 101]. Furthermore, phosphodiesterase inhibitors have been used as treatments for male infertility as they act as cAMP-level modulators [102]. Estrogen signaling is one of the essential pathways for normal spermatogenesis, testicular development, and sperm motility [103–105]. While the importance of enriched signaling pathways in fertility is clear, the role of all top-scored L-R pairs or highly influential nodes in the L-R interaction network in male infertility is not well understood. However, there is evidence to support their involvement in spermatogenesis and infertility. It has been reported that there is an association between *HLA-C* alleles and semen hyperviscosity and oligozoospermia [106], as well as a potential role in reproductive failures [107]. *CALM1*, *CALM2*, and *CALM3* are genes that encode for calcium signaling pathways, regulating many cellular processes, including spermatogenesis [108]. High *CALM1* expression is reported in women with polycystic ovary syndrome disorder [109]. *B2M*, a gene involved in immune responses and apoptosis triggered by an elevation in antigen presentation, was found to be up-regulated in Klinefelter syndrome patients [110]. GNAS is involved in G protein-coupled receptor signaling, and differentially methylated regions in *GNAS* may be associated with idiopathic male infertility [111]. Low methylation at *GNAS* has also been reported to be more prevalent in idiopathic infertile males, particularly in oligozoospermic males [112]. A case report showed that a *GNAS*-activating mutation caused a Leydig cell tumor and hypertestosteronemia [113]. The impact of stress on sperm function and *ADRB2* expression has been reported, indicating a reduction in spermatozoa functionality [114]. The *SLC9C2* gene product is localized to the head of mature mammalian sperm and is suggested to have an important role in male fertility [115]. A reduced expression of *CRHR1* in asthenozoospermic patients compared to controls with progressive sperm motility lower than 15% is reported [116]. Also, the role of CRHR1 in the autocrine/paracrine pathway of eutopic and ectopic endometrium has been demonstrated, which is suggested to potentially affect the pathogenesis of endometriosis and the infertility profile of affected women [117].

## Conclusion

In summary, this study integrated more than 26,000 cells from different testicular scRNA-seq datasets, from embryos to adults, to construct a testis transcriptional cell atlas, which grouped the cells into human FGC, infants, childhood, peri-puberty, and adults. Clustering, cell type assignments, DEG analysis, and enrichment analysis revealed three and two different types of Sertoli and Myoid cells, respectively, present in varying proportions across the five developmental stages. Additionally, two different SSC and Round SPT cell types were identified. The topological analysis of the WGCN of the testis transcriptional cell atlas highlighted important somatic genes in this network, including *RPL39*, *RPL10*, *RPL13A*, *FTH1*, *RPS2*, and *RPL18A*, which have been reported to be associated with infertility. Paracrine cell–cell communication also highlighted the effects of somatic cells as ligands on germ cells as receptors. The “Neuroactive ligand-receptor interaction,” “cAMP signaling pathway,” and “Estrogen signaling pathway” were found to be the most influential pathways. The top-scoring L-R pairs and more influential nodes in the cell–cell communication network were identified, and evidence to support their involvement in spermatogenesis and infertility suggests that they could serve as potential markers for infertility. Therefore, our study provides insights into the cellular heterogeneity of somatic and germ cells and identifies key genes in somatic cells that may impact germ cells during normal spermatogenesis, potentially playing significant roles in male infertility disorders. These findings may serve as a basis for further experimental research to explore the functions of these genes in male infertility.

### Supplementary Information

**Additional file 1**: **Supplementary figures and tables. Table S1. **Dataset information is listed in detail: Dataset names, the age of donors, scRNA-seq methods, GEO IDs, and number of cells collected for each dataset. **Fig. S1.** Representation of data and datasets in the UMAP space of integrated data. In the left panel, the cells of each dataset and in the right panel cells of each data are colored and shown in the low-dimensional UMAP space of integrated data. Each row in the left panel represents the sum of that row in the right panel. **Table S2.** Characteristics of clusters. The numbers of cells for each dataset, cluster, and cell type assignment for each cluster were specified. The rows related to somatic, SSC, SPC, and SPT are colored gray, blue, red, and green, respectively. **Fig. S2.** Expression pattern of Sertoli markers. **A** Gene expression patterns of Sertoli markers in the UMAP space. **B** Dot-plot presentation of markers expression. **Fig. S3.** Expression pattern of Leydig markers. **A** Gene expression patterns of Leydig markers in the UMAP space. **B** Dot-plot presentation of markers expression. ALD1H1 is a common marker for Sertoli and Leydig cells. **Fig. S4.** Expression pattern of Myoid markers. **A** Gene expression patterns of Myoid markers in the UMAP space. **B** Dot-plot presentation of markers expression. **Fig. S5.** Expression pattern of Macrophage markers. **A** Gene expression patterns of Macrophage markers in the UMAP space. **B** Dot-plot presentation of markers expression. **Fig. S6.** Expression pattern of Endothelial markers. **A** Gene expression patterns of Endothelial markers in the UMAP space. **B** Dot-plot presentation of markers expression. **Fig. S7.** Expression pattern of spermatogonia cell markers. **A** Gene expression patterns of spermatogonia cell markers in the UMAP space. **B** Dot-plot presentation of markers expression. **Fig. S8.** Expression pattern of spermatocyte cell markers. **A** Gene expression patterns of spermatocyte cell markers in the UMAP space. **B** Dot-plot presentation of markers expression. **Fig. S9.** Expression pattern of spermatid cell markers. **A** Gene expression patterns of spermatid cell markers in the UMAP space. **B** Dot-plot presentation of markers expression. **Fig. S10.** L-R interactions between somatic-germ cells. **A** Myoid-2 as ligand, Undifferentiated SSC, and Round SPT-1 as receptors, **B** macrophage as ligand, Undifferentiated SSC, and Round SPT-1 as receptors, **C** Sertoli-3 as ligand, Early round SPT, and Round SPT-1 as receptors.

**Additional file 2**: The differentially expressed genes for each of cell-type clusters

**Additional file 3**: Degree centrality and p-values for each node of WGCN of the integrated data

**Additional file 4**: Paracrine cell-cell communication among all cell types in scRNA-seq profiles

**Additional file 5**: The details of all somatic and germ cells L-R interactions

**Additional file 6**: Degree centrality in the weighted L-R interaction network

## Data Availability

The gene expression datasets analyzed in this study were extracted from Gene Expression Omnibus (GEO) and is available under Accession Numbers GSE86146, GSE124263, GSE120506, GSE134144, GSE109037, GSE106487. Codes are available at https://github.com/nasalehi/scRNAseq_testis_cell_atlas.

## Abbreviations

BP: Biological process
DEG: Differentially expressed gene
GEO: Gene Expression Omnibus
FGC: Fetal germ cell
L-R: Ligand-receptor
PCA: Principal component analysis
scRNA-seq: Single-cell RNA sequencing
SPC: Spermatocyte
SPT: Spermatid
SSC: Spermatogonial stem cells
WGCN: Weighted gene co-expression network
