# Supplementary material for: The construction of a testis transcriptional cell atlas from embryo to adult reveals various somatic cells and their molecular roles
Source: J Transl Med. 2023 Nov 27;21:859. doi: 10.1186/s12967-023-04722-2 (PMC10680190; doi:10.1186/s12967-023-04722-2)
Supplement: Supplementary file 1 — Additional file 1: Supplementary figures and tables. Table S1. Dataset information is listed in detail: Dataset names, the age of donors, scRNA-seq methods, GEO IDs, and number of cells collected for each dataset. Fig. S1. Representation of data and datasets in the UMAP space of integrated data. In the left panel, the cells of each dataset and in the right panel cells of each data are colored and shown in the low-dimensional UMAP space of integrated data. Each row in the left panel represents the sum of that row in the right panel. Table S2. Characteristics of clusters. The numbers of cells for each dataset, cluster, and cell type assignment for each cluster were specified. The rows related to somatic, SSC, SPC, and SPT are colored gray, blue, red, and green, respectively. Fig. S2. Expression pattern of Sertoli markers. A Gene expression patterns of Sertoli markers in the UMAP space. B Dot-plot presentation of markers expression. Fig. S3. Expression pattern of Leydig markers. A Gene expression patterns of Leydig markers in the UMAP space. B Dot-plot presentation of markers expression. ALD1H1 is a common marker for Sertoli and Leydig cells. Fig. S4. Expression pattern of Myoid markers. A Gene expression patterns of Myoid markers in the UMAP space. B Dot-plot presentation of markers expression. Fig. S5. Expression pattern of Macrophage markers. A Gene expression patterns of Macrophage markers in the UMAP space. B Dot-plot presentation of markers expression. Fig. S6. Expression pattern of Endothelial markers. A Gene expression patterns of Endothelial markers in the UMAP space. B Dot-plot presentation of markers expression. Fig. S7. Expression pattern of spermatogonia cell markers. A Gene expression patterns of spermatogonia cell markers in the UMAP space. B Dot-plot presentation of markers expression. Fig. S8. Expression pattern of spermatocyte cell markers. A Gene expression patterns of spermatocyte cell markers in the UMAP space. B Dot-plot presentation of markers ex [file 12967_2023_4722_MOESM1_ESM.docx]

**The Construction of a Testis Transcriptional Cell Atlas from Embryo to Adult Reveals Various Somatic Cells and Their Molecular Roles**

Najmeh Salehi^1*^, Mehdi Totonchi^2*^

^1^ School of Biological Science, Institute for Research in Fundamental Sciences (IPM), Tehran, Iran.

^2^ Department of Genetics, Reproductive Biomedicine Research Center, Royan Institute for Reproductive Biomedicine, ACECR, Tehran, Iran.

^*^ Correspondence: [nsalehi@ipm.ir](mailto:nsalehi@ipm.ir), [m.totonchi@royaninstitute.org](mailto:m.totonchi@royaninstitute.org)

| **Dataset Name** | **Age** | **scRNA-seq Method** | **GEO ID** | **Cells** |
| --- | --- | --- | --- | --- |
| Fetal | 4-25 W_e_ | Smart-seq2 | GSE86146 | 1,187 |
| Infancy | 2 Days | 10x Genomics | GSE124263 | 3,634 |
|  | 7 Days | 10x Genomics | GSE124263 | 5,155 |
| Childhood | 1 Year | 10x Genomics | GSE120506 | 1,341 |
|  | 7 Year | 10x Genomics | GSE134144 | 1,968 |
|  | 11 Year | 10x Genomics | GSE134144 | 4,176 |
| Peri-puberty | 13 Year | 10x Genomics | GSE134144 | 4,051 |
|  | 14 Year | 10x Genomics | GSE134144 | 2,722 |
| Adulthood | 41.6 ± 1.3yr | 10x Genomics | GSE109037 | 7,134 |
|  | 38.5± 9.6yr | Smart-seq2 | GSE106487 | 3,046 |

**Table S1.** Dataset information is listed in detail: Dataset names, the age of donors, scRNA-seq methods, GEO IDs, and number of cells collected for each dataset.


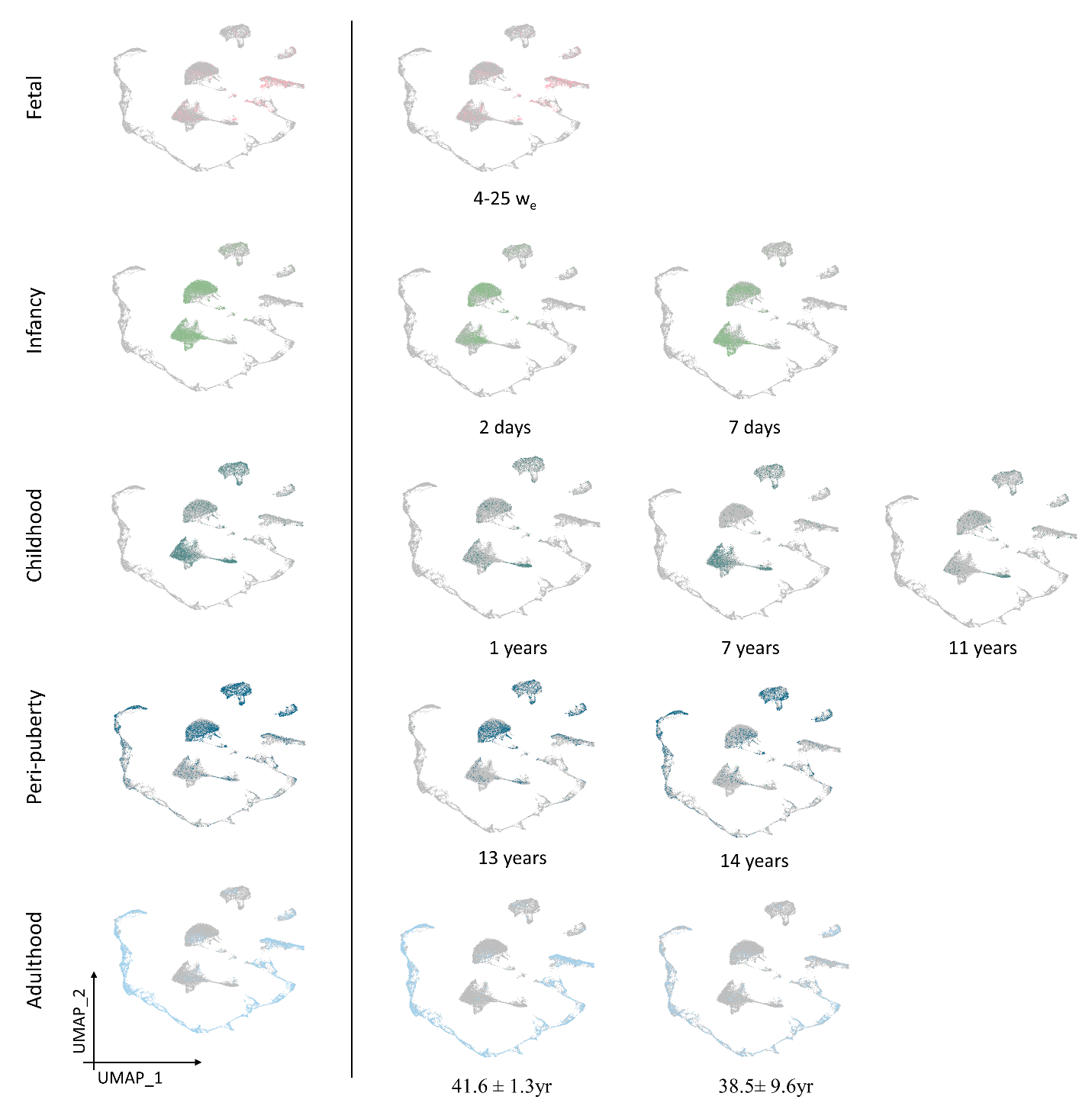


**Fig. S1.** **Representation of data and datasets in the UMAP space of integrated data.** In the left panel, the cells of each dataset and in the right panel cells of each data are colored and shown in the low-dimensional UMAP space of integrated data. Each row in the left panel represents the sum of that row in the right panel.


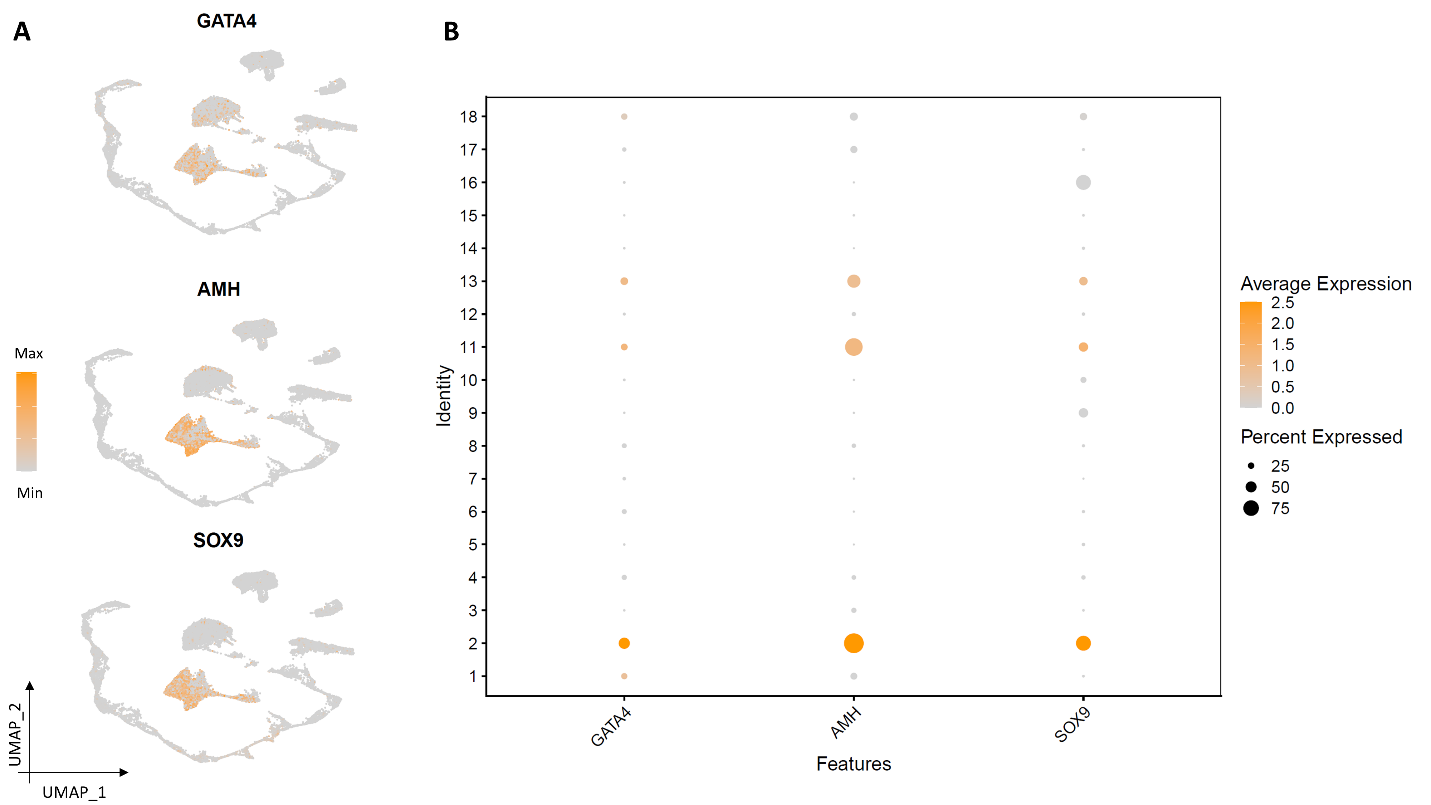


**Fig. S2.** **Expression pattern of Sertoli markers.** (A) Gene expression patterns of Sertoli markers in the UMAP space. (B) Dot-plot presentation of markers expression.


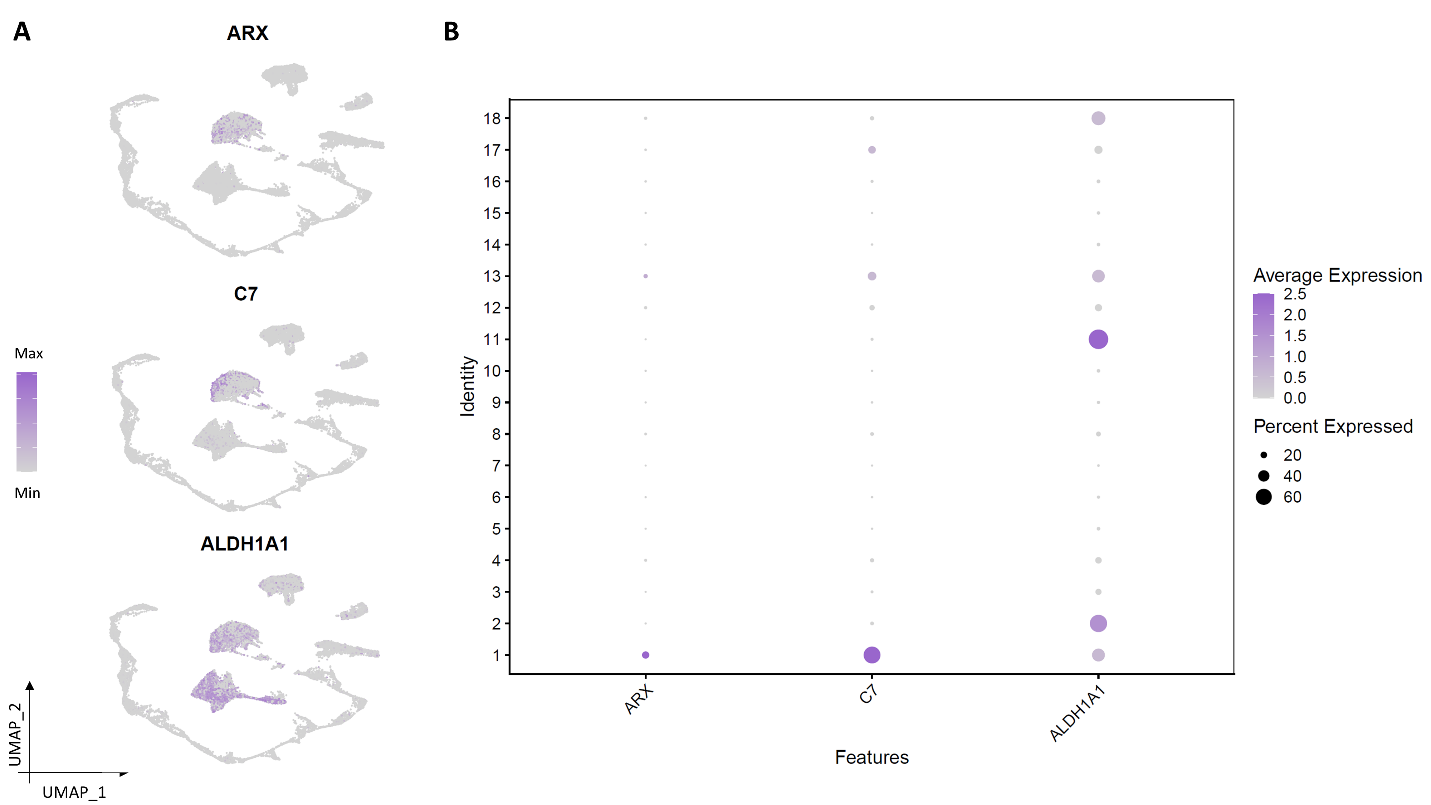


**Fig. S3.** **Expression pattern of Leydig markers.** (A) Gene expression patterns of Leydig markers in the UMAP space. (B) Dot-plot presentation of markers expression. ALD1H1 is a common marker for Sertoli and Leydig cells.


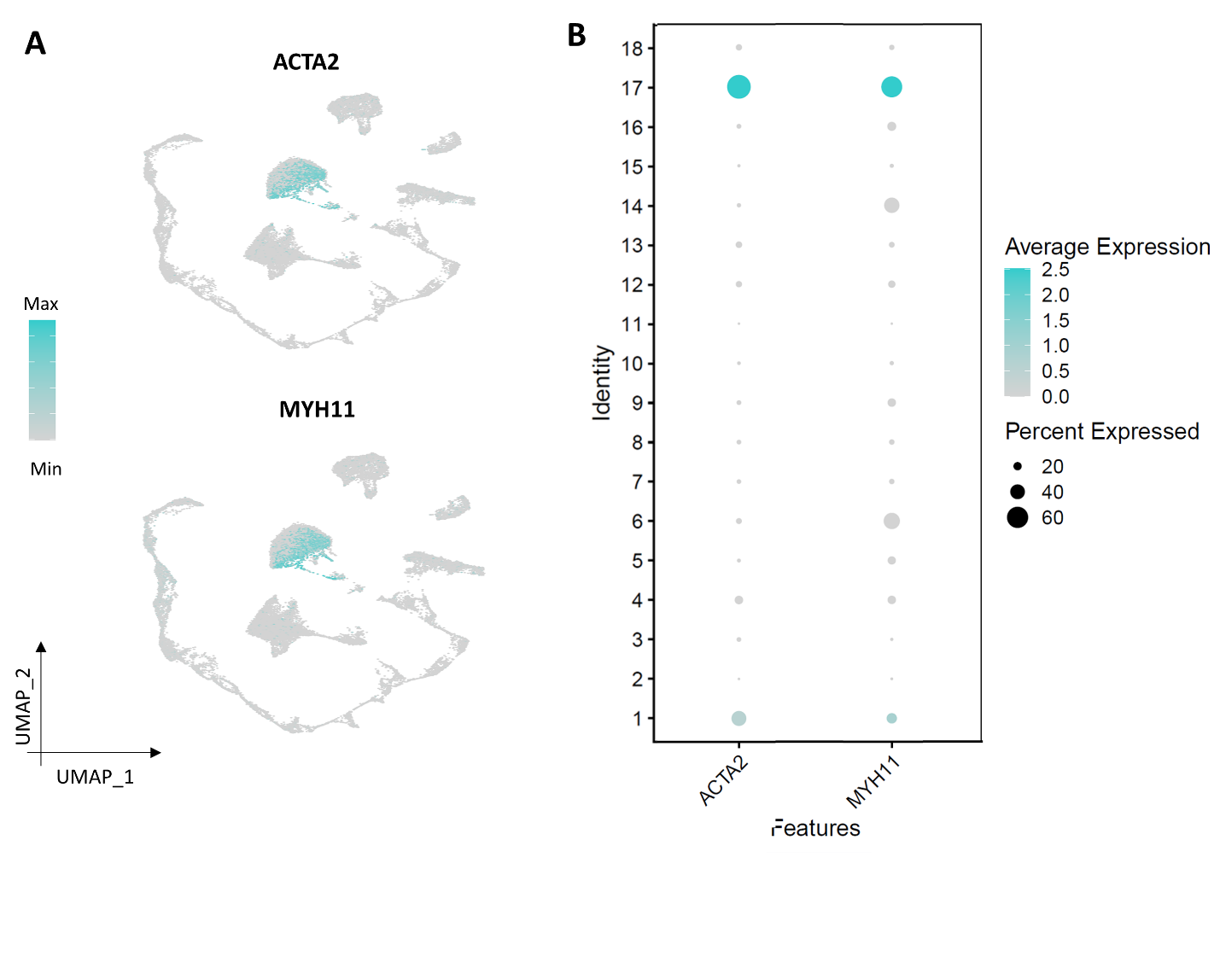
**Fig. S4.** **Expression pattern of Myoid markers.** (A) Gene expression patterns of Myoid markers in the UMAP space. (B) Dot-plot presentation of markers expression.


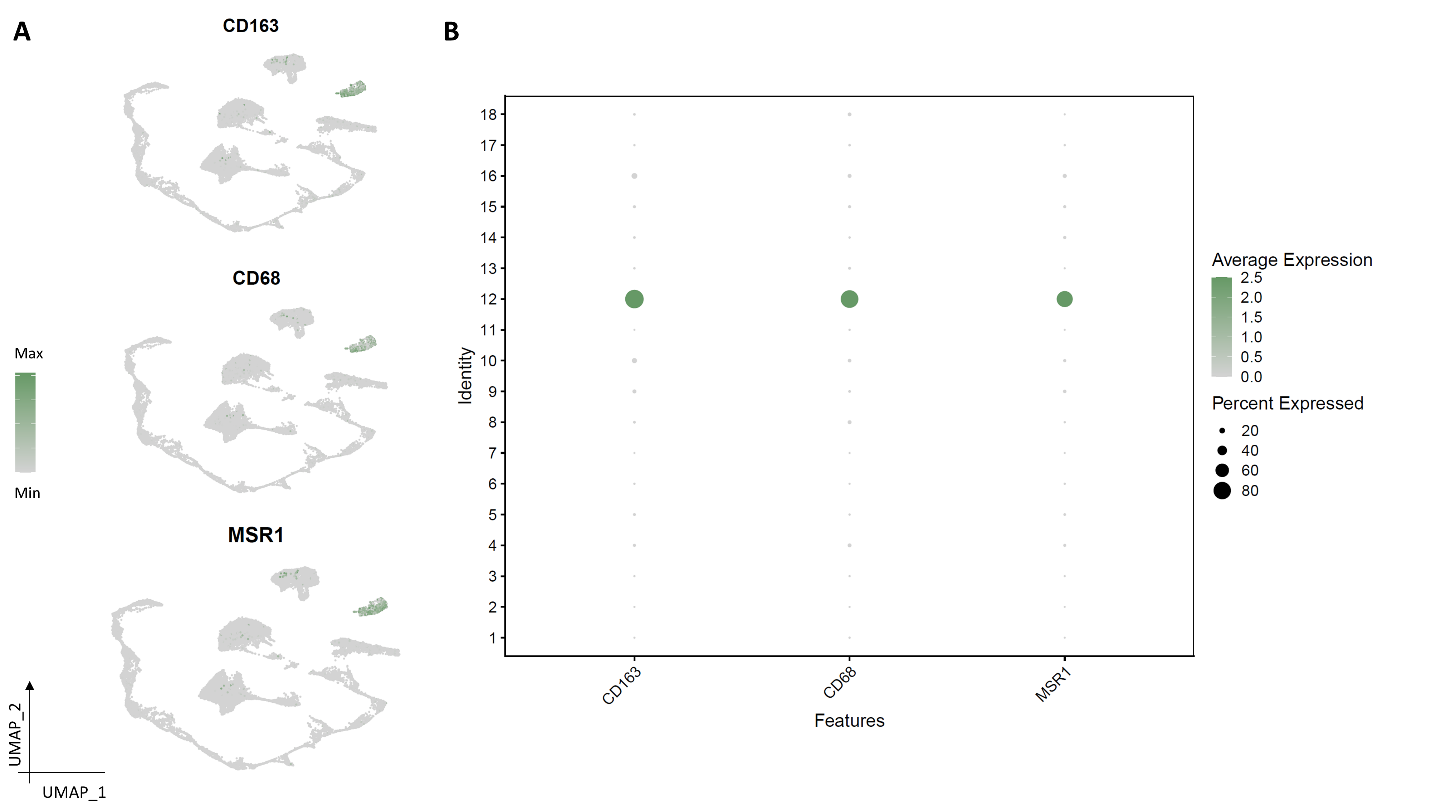


**Fig. S5.** **Expression pattern of Macrophage markers.** (A) Gene expression patterns of Macrophage markers in the UMAP space. (B) Dot-plot presentation of markers expression.


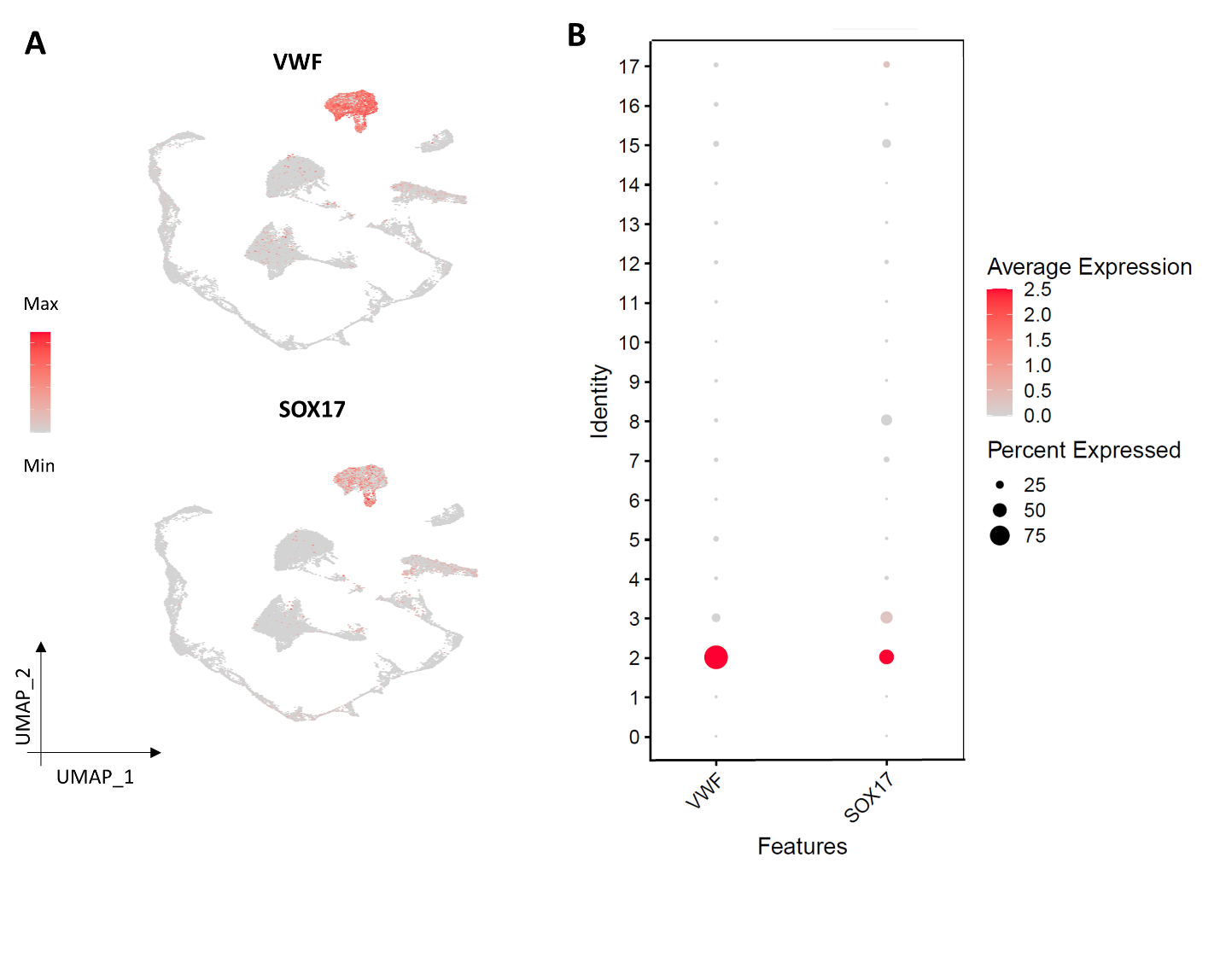


**Fig. S6.** **Expression pattern of Endothelial markers.** (A) Gene expression patterns of Endothelial markers in the UMAP space. (B) Dot-plot presentation of markers expression.


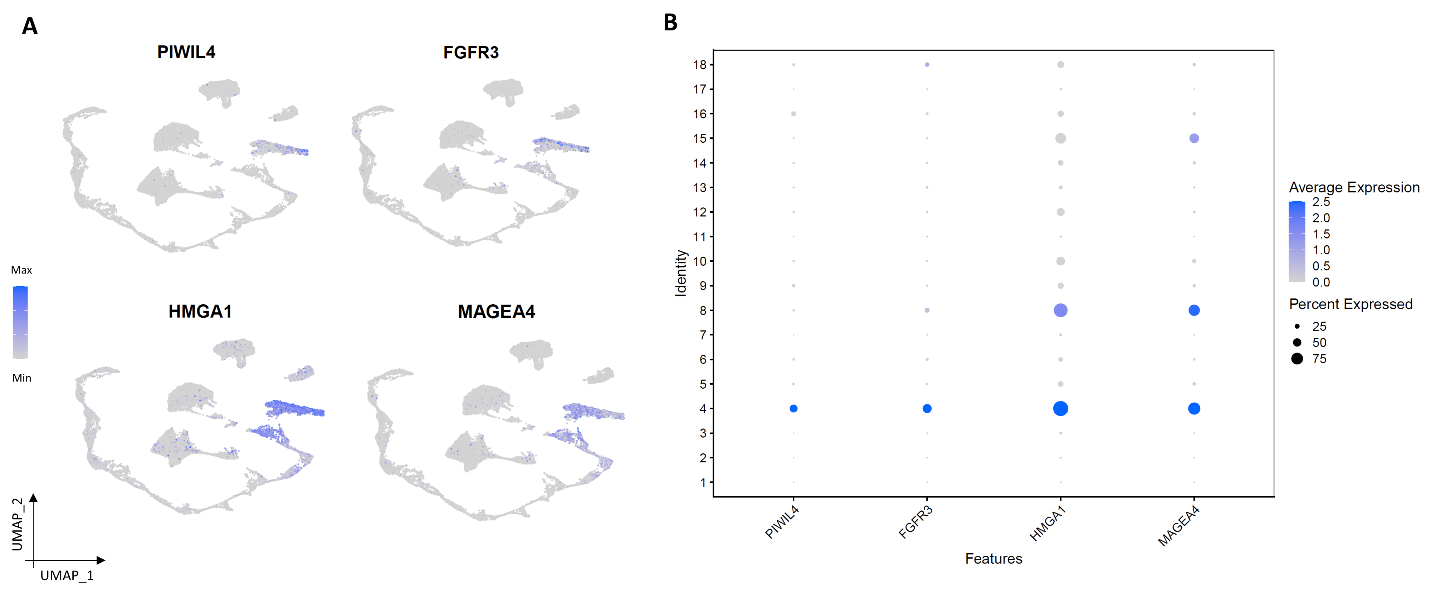


**Fig. S7.** **Expression pattern of spermatogonia cell markers.** (A) Gene expression patterns of spermatogonia cell markers in the UMAP space. (B) Dot-plot presentation of markers expression.


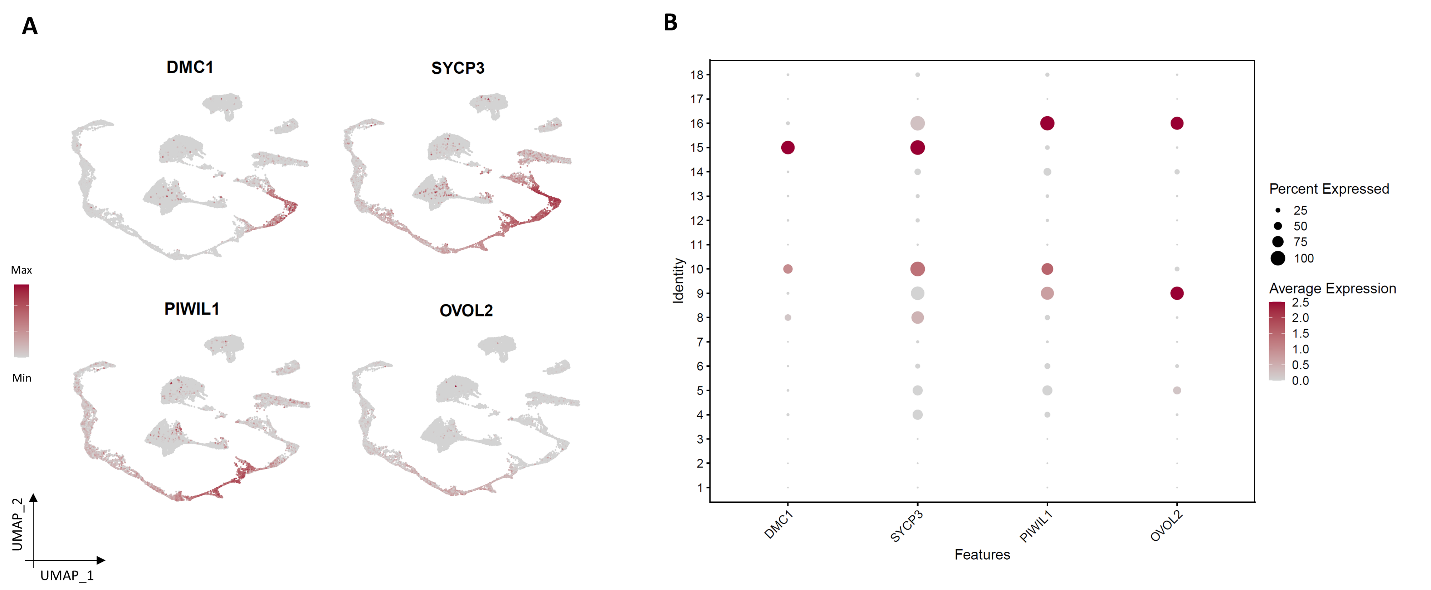


**Fig. S8.** **Expression pattern of spermatocyte cell markers.** (A) Gene expression patterns of spermatocyte cell markers in the UMAP space. (B) Dot-plot presentation of markers expression.


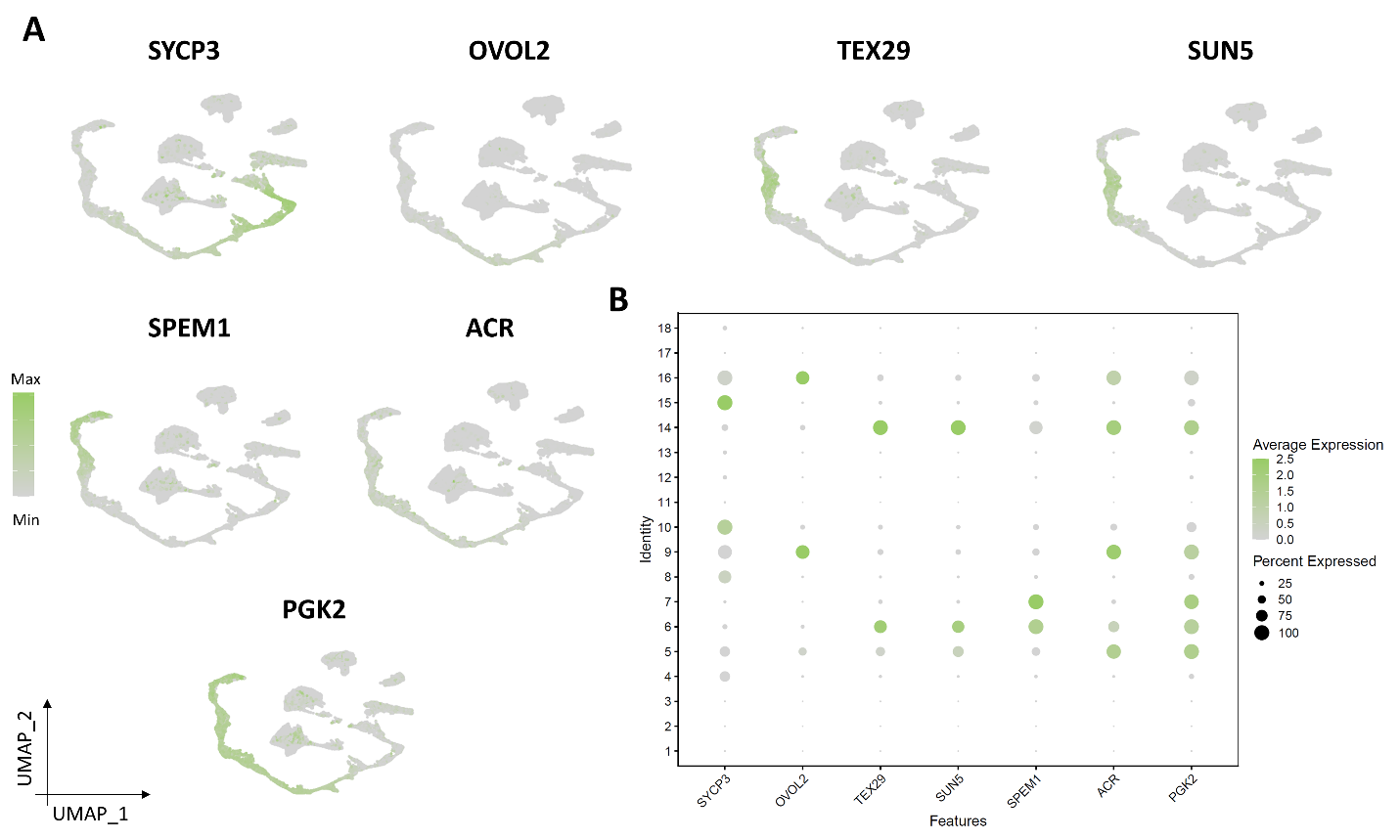


**Fig. S9.** **Expression pattern of spermatid cell markers.** (A) Gene expression patterns of spermatid cell markers in the UMAP space. (B) Dot-plot presentation of markers expression.

**Table S2.** **Characteristics of clusters.** The numbers of cells for each dataset, cluster, and cell type assignment for each cluster were specified. The rows related to somatic, SSC, SPC, and SPT are colored gray, blue, red, and green, respectively.

| **Clusters** | **Fetal** | **Infancy** | **Childhood** | **Peri-puberty** | **Adulthood** | **Cell Type** |
| --- | --- | --- | --- | --- | --- | --- |
| 1 | 106 | 2998 | 524 | 1453 | 185 | Leydig |
| 2 | 187 | 2790 | 1441 | 208 | 71 | Sertoli-1 |
| 3 | 26 | 261 | 888 | 1316 | 92 | Endothelial |
| 4 | 484 | 21 | 107 | 217 | 1439 | Undifferentiated SSC |
| 5 | 0 | 0 | 3 | 112 | 1747 | Early round SPT |
| 6 | 0 | 0 | 1 | 299 | 1180 | Round SPT-2 |
| 7 | 0 | 0 | 1 | 362 | 741 | Elongating SPT |
| 8 | 160 | 68 | 31 | 83 | 761 | Differentiating SSC |
| 9 | 0 | 0 | 3 | 51 | 1047 | Diplotene SPC |
| 10 | 0 | 0 | 6 | 53 | 1042 | Zygotene SPC |
| 11 | 41 | 33 | 725 | 59 | 10 | Sertoli-3 |
| 12 | 59 | 31 | 70 | 409 | 202 | Macrophage |
| 13 | 79 | 207 | 143 | 75 | 85 | Sertoli-2 |
| 14 | 0 | 0 | 1 | 67 | 510 | Round SPT-1 |
| 15 | 0 | 0 | 6 | 36 | 474 | Leptotene SPC |
| 16 | 0 | 0 | 0 | 15 | 403 | Pachytene SPC |
| 17 | 5 | 109 | 24 | 83 | 11 | Myoid-1 |
| 18 | 21 | 57 | 11 | 8 | 8 | Myoid-2 |


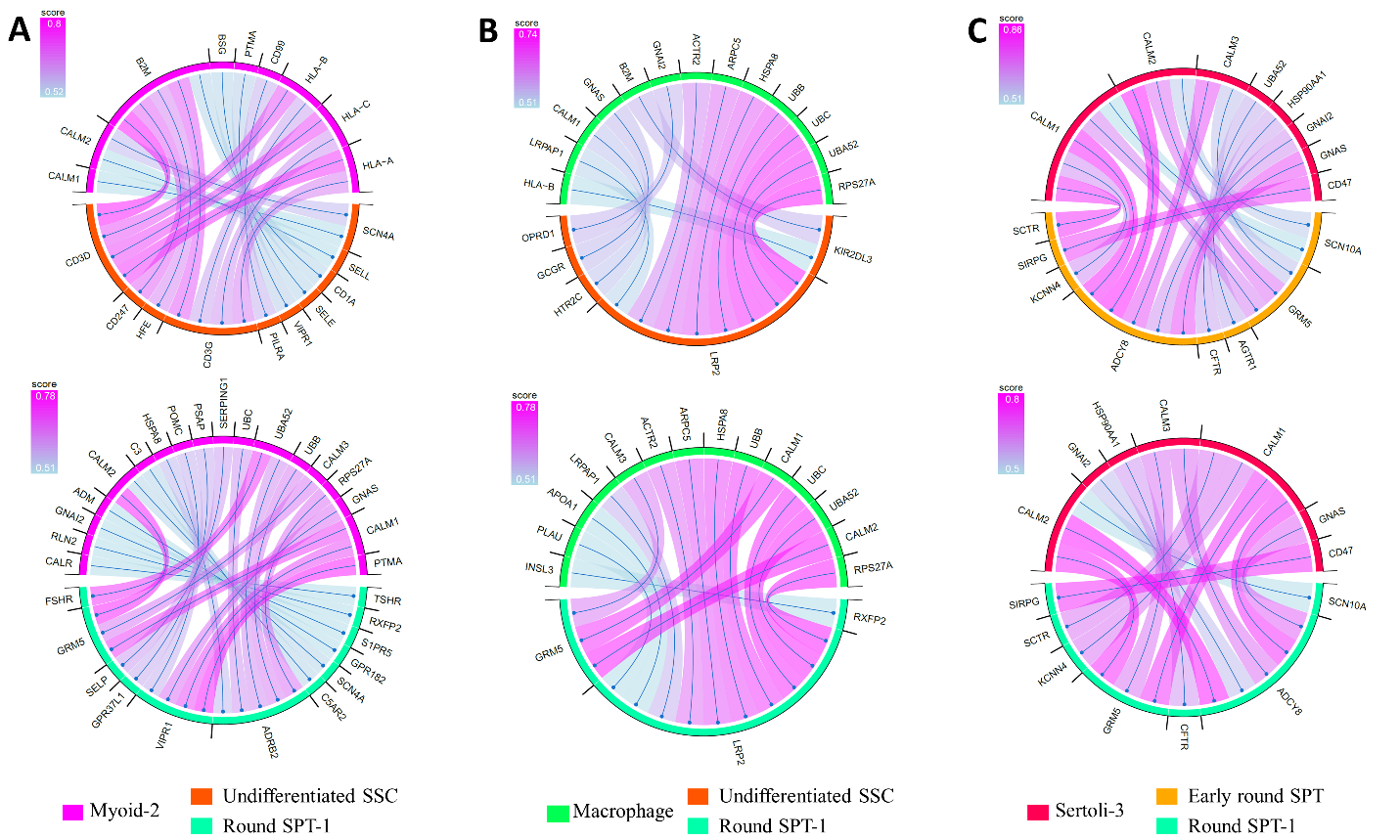


**Fig. S10.** **L-R interactions between somatic-germ cells.** (A) Myoid-2 as ligand, Undifferentiated SSC, and Round SPT-1 as receptors, (B) Macrophage as ligand, Undifferentiated SSC, and Round SPT-1 as receptors, (C) Sertoli-3 as ligand, Early round SPT, and Round SPT-1 as receptors.
